# Supplementary material for: Association between early gestation passive smoke exposure and neonatal size among self-reported non-smoking women by race/ethnicity: A cohort study
Source: PLoS One. 2021 Nov 18;16(11):e0256676. doi: 10.1371/journal.pone.0256676 (PMC8601432; doi:10.1371/journal.pone.0256676)
Supplement: S1 Fig — (DOCX) [file pone.0256676.s001.docx]

**S1 Fig. Participant flowchart.**

A

**Initial population in Fetal Growth Studies-Singletons**

N=2334

**Study cohort for main analyses (birthweight)**

**(**N=2055)

**Exclusion criteria (n= 279):**

- Ineligible after enrollment: 14 (5.0%)
- No live birth: 186 (66.7%)
- Did not provide blood sample at baseline: 28 (10.0%)
- Insufficient sample for analysis (nicotine/cotinine measurement) available: 5 (1.8%)
- Did not consent to use of blood sample: 46 (16.5%)

A+B

**Skinfold analyses**

N=1806

**%fat mass analyses**

N=1649

A + B +C +D

A: Missing time to study exam after birth (n=120)

B: Incorrect calipers used (n= 129)

C: <37 weeks, <2000g (n= 99; outside validated range per formula to calculate %fat)

D: Negative and missing values of % fat mass (n=58)

**Study exam (birthweight, length, circumferences)**

N=1935
