# Supplementary material for: Association between early gestation passive smoke exposure and neonatal size among self-reported non-smoking women by race/ethnicity: A cohort study
Source: PLoS One. 2021 Nov 18;16(11):e0256676. doi: 10.1371/journal.pone.0256676 (PMC8601432; doi:10.1371/journal.pone.0256676)
Supplement: S3 Fig — (DOCX) [file pone.0256676.s003.docx]

**S3 Fig. Distribution of plasma nicotine concentrations above the LOQ^a^ overall and by race/ethnicity, NICHD Fetal Growth Studies-Singletons 2009-2013.**

^a^LOQ_nicotine_ = 0.13 ng/mL.

Abbreviations: LOQ, limit of quantification; NICHD, *Eunice Kennedy Shriver* National Institute of Child Health and Human Development; PI, Pacific Islander.
