# Supplementary material for: Association between early gestation passive smoke exposure and neonatal size among self-reported non-smoking women by race/ethnicity: A cohort study
Source: PLoS One. 2021 Nov 18;16(11):e0256676. doi: 10.1371/journal.pone.0256676 (PMC8601432; doi:10.1371/journal.pone.0256676)
Supplement: S1 Table — (DOCX) [file pone.0256676.s004.docx]

**S1 Table. Comparison of plasma biomarker concentration among included versus excluded women.**

|  | **Included (n=2055)** | **Excluded (n=190)^a^** | **p-value^b^** |
| --- | --- | --- | --- |
| **Cotinine (ng/mL)** | | | |
| Median (IQR) | 0.009 (0.0, 0.037) | 0.010 (0.0, 0.042) | 0.836 |
| Non-smoker; n (%)^c^ | 1993 (97.0) | 184 (96.9) | 0.826 |
| Passive smoker; n (%)^c^ | 62 (3.0) | 6 (3.1) |  |
| %≥LOQ; n (%)^d^ | 450 (23.7) | 45 (21.9) | 0.570 |
| **Nicotine (ng/mL)** | | | |
| Median (IQR) | -0.007 (-0.039, 0.040) | -0.005 (-0.038, 0.031) | 0.915 |
| %≥LOQ; n (%)^e^ | 282 (13.7) | 25 (13.2) | 0.828 |

^~~a~~^7 for miscarriage; 13 for fetal death; 7 for voluntary termination of pregnancy; 163 unknown outcomes.

^b^Kruskal-Wallis nonparametric tests conducted to compare medians of biomarker concentration for continuous variables; Chi-square test.

^c^Non-smoker: unexposed/typical passive smoke exposure: <1 ng/mL; Passive smoker: ≥1 ng/mL cotinine.

^d^LOQ_cotinine_ = 0.05 ng/mL.

^e^LOQ_nicotine_ = 0.13 ng/mL.

Abbreviations: IQR, inter-quartile range; LOQ, limit of quantification.
