# Supplementary material for: Association between early gestation passive smoke exposure and neonatal size among self-reported non-smoking women by race/ethnicity: A cohort study
Source: PLoS One. 2021 Nov 18;16(11):e0256676. doi: 10.1371/journal.pone.0256676 (PMC8601432; doi:10.1371/journal.pone.0256676)
Supplement: S2 Table — (DOCX) [file pone.0256676.s005.docx]

**S2 Table. Interaction by race/ethnicity in the plasma biomarker concentrations-neonatal anthropometrics association among non-smoking pregnant women.^a^**

| **Neonatal anthropometric measure** | **Continuous plasma concentration^b^** | | **Categorical** | | |
| --- | --- | --- | --- | --- | --- |
|  | **Cotinine** | **Nicotine** | **Smoking per cotinine^c^** | **LOQ_cotinine_^d^** | **LOQ_nicotine_^e^** |
| **Unadjusted model^f^** | | | | | |
| *Non-skeletal measures* | | | | | |
| Birthweight | **0.014** | **0.013** | 0.140 | 0.155 | **0.017** |
| Mid-upper arm circumference | 0.219 | **0.085** | 0.448 | **0.069** | 0.666 |
| Abdominal circumference | **0.033** | **0.007** | **0.064** | 0.574 | **0.021** |
| Mid-upper thigh circumference | **0.040** | **0.014** | **0.070** | **0.083** | **0.034** |
| Subscapular skinfold | **0.015** | **0.019** | **0.088** | 0.392 | 0.127 |
| Triceps skinfold | **0.048** | **0.052** | **0.082** | 0.370 | 0.222 |
| Abdominal flank skinfold | 0.842 | 0.59 | 0.786 | **0.043** | **0.010** |
| Anterior thigh skinfold | **0.004** | **0.010** | **0.054** | **0.047** | **0.017** |
| Percent fat mass | **0.086** | 0.300 | 0.111 | 0.103 | 0.161 |
| *Skeletal measures* | | | | | |
| Exam length | 0.199 | 0.809 | 0.303 | 0.479 | 0.944 |
| Head circumference | 0.303 | **0.088** | 0.656 | 0.156 | **0.096** |
| *Clinical outcomes^g^* |  |  |  |  |  |
| Low birthweight (<2500 g) | 0.150 | 0.806 | n/a | n/a | n/a |
| Macrosomia (>4000 g) | 0.277 | 0.433 | n/a | n/a | n/a |
| **Adjusted model^g^** | | | | | |
| *Non-skeletal measures* | | | | | |
| Birthweight | **0.0344** | **0.0388** | 0.1949 | 0.1951 | **0.0430** |
| Mid-upper arm circumference | 0.3579 | 0.1504 | 0.4989 | **0.0965** | 0.8018 |
| Abdominal circumference | **0.0446** | **0.0154** | **0.0667** | 0.7144 | **0.0403** |
| Mid-upper thigh circumference | **0.0665** | **0.0316** | **0.0779** | 0.1055 | **0.0679** |
| Subscapular skinfold | **0.0139** | **0.0378** | **0.0755** | 0.2908 | 0.2175 |
| Triceps skinfold | **0.0553** | **0.0812** | **0.0638** | 0.3524 | 0.2752 |
| Abdominal flank skinfold | 0.8848 | 0.6560 | 0.7434 | **0.0512** | **0.0116** |
| Anterior thigh skinfold | **0.0044** | **0.0136** | **0.0452** | **0.0468** | **0.0248** |
| Percent fat mass | 0.1108 | 0.3587 | **0.0813** | 0.1578 | 0.2161 |
| *Skeletal measures* | | | | | |
| Exam length | 0.4009 | 0.9130 | 0.4757 | 0.2571 | 0.8208 |
| Head circumference | 0.5973 | 0.2338 | 0.8736 | 0.1322 | 0.2104 |
| *Clinical outcomes^g^* |  |  |  |  |  |
| Low birthweight (<2500 g) | 0.2216 | 0.7572 | n/a | n/a | n/a |
| Macrosomia (>4000 g) | 0.5857 | 0.5736 | n/a | n/a | n/a |

^a^Values reported in table are p-values from Type III sums of squares for biomarker x race/ethnicity interaction term (*P_interaction_*; global); *P_interaction_*<0.1 considered statistically significant; derived from generalized linear models unless otherwise noted.

^b^Plasma concentrations of cotinine and nicotine (ng/mL) were log-transformed (log[1+value]) then rescaled by their standard deviation for analysis.

^c^Passive smoker (≥1 ng/mL) vs non-smoker (reference; <1 ng/mL).

^d^≥LOQ vs < LOQ (reference; LOQ_cotinine_ = 0.05 ng/mL).

^e^≥LOQ vs < LOQ (reference; LOQ_nicotine_ = 0.13 ng/mL).

^f^Adjusted for time to exam only (except birthweight measures performed at birth).

^g^Based on logistic regression models; each model separately in comparison to normal birthweight group (2500-4000 g); p-values from Wald chi-squared joint test for biomarker x race/ethnicity interaction term (global); p<0.1 considered statistically significant; not conducted for categorical exposures due to sparse data.

^h^Adjusted for maternal age, infant sex, maternal height, weight, education, parity, and time to exam (except birthweight measures performed at birth).

Abbreviations: LOQ, limit of quantification.

**BOLD: Statistically significant differences in the association between biomarker concentration and neonatal anthropometric measure by race/ethnicity (*P_interaction_*<0.1)**.
