# Supplementary material for: Association between early gestation passive smoke exposure and neonatal size among self-reported non-smoking women by race/ethnicity: A cohort study
Source: PLoS One. 2021 Nov 18;16(11):e0256676. doi: 10.1371/journal.pone.0256676 (PMC8601432; doi:10.1371/journal.pone.0256676)
Supplement: S3 Table — (DOCX) [file pone.0256676.s006.docx]

**S3 Table. Plasma biomarker concentration-neonatal anthropometrics associations by race/ethnicity among non-smoking pregnant women (unadjusted models).^a^**

| **Biomarker and smoking status** | **Non-skeletal measures** | | | | | | | | | **Skeletal measures (cm; n=1935)** | |
| --- | --- | --- | --- | --- | --- | --- | --- | --- | --- | --- | --- |
|  | **Birthweight (g; n=2055)** | **Circumferences (cm; n=1935)** | | | **Skinfolds (mm; n=1806)^b^** | | | | **Percent fat mass (n=1649)^c^** | **Exam length** | **Head circumfer-ence** |
|  |  | **Mid-upper arm** | **Abdominal** | **Mid-upper thigh** | **Subscap-ular** | **Triceps** | **Abdominal flank** | **Anterior thigh** |  |  |  |
| **CONTINUOUS BIOMARKER ASSESSMENT** | | | | | | | | | | | |
| ***Cotinine*** | | | | | | | | | | | |
| White | **50.7 (-43.1, 144.6)** | 0.17 (-0.07, 0.41) | **0.19 (-0.23, 0.61)** | **0.23 (-0.16, 0.61)** | **0.36 (0.12, 0.60)** | **0.31 (0.04, 0.57)** | 0.09 (-0.18, 0.36) | **0.64 (0.28, 1.0)** | **0.46 (-0.25, 1.2)** | -0.06 (-0.55, 0.42) | 0.03 (-0.25, 0.32) |
| Asian/PI | **-62.8 (-469.9, 344.2)** | 0.47 (-0.59, 1.5) | **-1.2 (-3.1, 0.67)** | **0.15 (-1.5, 1.8)** | **-0.76 (-1.8, 0.31)** | **-0.68 (-1.9, 0.50)** | -0.37 (-1.6, 0.82) | **-0.31 (-1.9, 1.3)** | **-2.9 (-6.0, 0.29)** | 0.71 (-1.4, 2.8) | -0.13 (-1.4, 1.1) |
| Hispanic | **68.2 (-67.3, 203.6)** | -0.04 (-0.39, 0.31) | **0.35 (-0.27, 0.96)** | **0.38 (-0.18, 0.94)** | **-0.09 (-0.44, 0.26)** | **0.41 (0.02, 0.80)** | 0.03 (-0.36, 0.42) | **0.54 (0.01, 1.1)** | **0.09 (-0.93, 1.1)** | 0.62 (-0.08, 1.3) | 0.00 (-0.41, 0.42) |
| Black | **-104.4 (-162.8, -46.0)** | -0.10 (-0.25, 0.05) | **-0.37 (-0.63, -0.10)** | **-0.30 (-0.54, -0.06)** | **-0.10 (-0.33, 0.14)** | **-0.08 (-0.33, 0.18)** | -0.03 (-0.29, 0.23) | **-0.23 (-0.57,0.12)** | **-0.47 (-1.2, 0.21)** | -0.17 (-0.48, 0.13) | -0.26 (-0.44, -0.08) |
| ***Nicotine*** | | | | | | | | | | | |
| White | **265.8 (28.1, 503.6)** | **0.53 (-0.08, 1.1)** | **0.99 (-0.08, 2.1)** | **0.95 (-0.02, 1.9)** | **1.4 (0.79, 2.0)** | **1.2 (0.53, 1.9)** | 1.0 (0.35, 1.7) | **2.4 (1.4, 3.3)** | 2.8 (1.0, 4.6) | -0.33 (-1.6, 0.90) | **0.50 (-0.23, 1.2)** |
| Asian/PI | **188.6 (-330.3, 707.5)** | **-0.86 (-2.2, 0.48)** | **-0.79 (-3.2, 1.6)** | **-0.70 (-2.8, 1.4)** | **1.0 (-0.32, 2.4)** | **0.19 (-1.3, 1.7)** | 0.66 (-0.85, 2.2) | **0.89 (-1.1, 2.9)** | 3.2 (-0.83, 7.2) | -1.0 (-3.7, 1.7) | **0.54 (-1.1, 2.1)** |
| Hispanic | **123.9 (-166.8, 414.5)** | **-0.26 (-1.0, 0.49)** | **0.53 (-0.79, 1.9)** | **-0.36 (-1.6, 0.83)** | **0.19 (-0.56, 0.94)** | **0.97 (0.13, 1.8)** | 0.28 (-0.56, 1.1) | **2.0 (0.83, 3.1)** | 1.1 (-1.2, 3.3) | 0.36 (-1.1, 1.9) | **0.33 (-0.57, 1.2)** |
| Black | **-167.8 (-318.0, -17.7)** | **-0.32 (-0.70, 0.07)** | **-1.1 (-1.8, -0.40)** | **-0.95 (-1.6, -0.34)** | **0.08 (-0.63, 0.80)** | **-0.18 (-0.97, 0.61)** | 0.79 (-0.00, 1.6) | **0.05 (-1.0, 1.1)** | 0.47 (-1.6, 2.6) | -0.29 (-1.1, 0.49) | **-0.47 (-0.93, -0.01)** |
| **CUT-POINT ANALYSIS** | | | | | | | | | | | |
| ***Cotinine*** | | | | | | | | | | | |
| *Any smoking^d^* | | | | | | | | | | | |
| White | -3.3 (-344.9, 338.3) | 0.59 (-0.28, 1.5) | **0.22 (-1.3, 1.8)** | **0.63 (-0.76, 2.0)** | **0.71 (-0.17, 1.6)** | **0.31 (-0.65, 1.3)** | -0.04 (-1.0, 0.93) | **1.3 (0.02, 2.7)** | 0.38 (-2.2, 2.9) | -0.67 (-2.4, 1.1) | -0.22 (-1.3, 0.81) |
| Asian/PI | -481.7 ( -1162, 198.4) | -0.05 (-1.8, 1.7) | **-2.9 (-6.0, 0.13)** | **-1.0 (-3.8, 1.7)** | **-1.3 (-3.1, 0.43)** | **-1.8 (-3.7, 0.12)** | -0.50 (-2.4, 1.4) | **-2.0 (-4.6, 0.66)** | -6.5 (-11.6, -1.4) | 0.33 (-3.2, 3.8) | -0.14 (-2.2, 1.9) |
| Hispanic | 42.0 (-351.7, 435.7) | -0.13 (-1.2, 0.97) | **0.64 (-1.3, 2.6)** | **1.0 (-0.72, 2.8)** | **0.20 (-0.90, 1.3)** | **1.2 (-0.06, 2.4)** | 0.55 (-0.68, 1.8) | **1.1 (-0.62, 2.7)** | 0.26 (-3.0, 3.5) | 1.4 (-0.83, 3.6) | 0.04 (-1.3, 1.3) |
| Black | -322.2 (-470.4,-174.0) | -0.20 (-0.59, 0.19) | **-1.3 (-2.0, -0.59)** | **-0.88 (-1.5, -0.26)** | **-0.44 (-1.1, 0.18)** | **0.09 (-0.61, 0.78)** | -0.05 (-0.75, 0.65) | **-0.32 (-1.3, 0.63)** | -0.71 (-2.6, 1.2) | -0.82 (-1.6, -0.03) | -0.68 (-1.1, -0.22) |
| **Biomarker and smoking status** | **Non-skeletal measures** | | | | | | | | | **Skeletal measures (cm; n=1935)** | |
|  | **Birthweight (g; n=2055)** | **Circumferences (cm; n=1935)** | | | **Skinfolds (mm; n=1806)^b^** | | | | **Percent fat mass (n=1649)^c^** | **Exam length** | **Head circumfer-ence** |
|  |  | **Mid-upper arm** | **Abdominal** | **Mid-upper thigh** | **Subscap-ular** | **Triceps** | **Abdominal flank** | **Anterior thigh** |  |  |  |
| *≥LOQ^e^* | | | | | | | | | | | |
| White | 46.3 (-89.3, 181.8) | **0.06 (-0.29, 0.41)** | -0.04 (-0.66, 0.58) | **-0.01 (-0.57, 0.55)** | -0.04 (-0.40, 0.32) | 0.08 (-0.32, 0.48) | **-0.12 (-0.52, 0.28)** | **0.40 (-0.14,0.95)** | -0.10 (-1.2, 1.0) | -0.18 (-0.90, 0.54) | 0.07 (-0.35, 0.49) |
| Asian/PI | 33.0 (-104.9, 170.8) | **0.33 (-0.04, 0.70)** | 0.29 (-0.36, 0.94) | **0.58 (-0.01, 1.2)** | -0.34 (-0.71, 0.04) | 0.20 (-0.21, 0.61) | **-0.29 (-0.71, 0.12)** | **0.18 (-0.39,0.74)** | -0.27 (-1.4, 0.84) | 0.16 (-0.57, 0.90) | 0.11 (-0.33, 0.55) |
| Hispanic | -45.6 (-158.7, 67.4) | **-0.08 (-0.38, 0.22)** | -0.00 (-0.53, 0.53) | **-0.22 (-0.70, 0.26)** | 0.08 (-0.22, 0.39) | 0.17 (-0.16, 0.51) | **0.34 (0.00, 0.68)** | **0.64 (0.18, 1.1)** | 0.73 (-0.18, 1.7) | -0.50 (-1.1, 0.11) | -0.39 (-0.75, -0.03) |
| Black | -110.4 (-195.3, -25.5) | **-0.23 (-0.46, -0.01)** | -0.24 (-0.64, 0.15) | **-0.30 (-0.66, 0.06)** | -0.02 (-0.30, 0.25) | -0.17 (-0.47, 0.13) | **-0.25 (-0.55, 0.05)** | **-0.22 (-0.63,0.19)** | -0.83 (-1.7, 0.00) | 0.03 (-0.43, 0.48) | -0.31 (-0.57, -0.04) |
| ***Nicotine*** | | | | | | | | | | | |
| *≥LOQ^f^* | | | | | | | | | | | |
| White | **137.9 (25.2, 250.6)** | 0.17 (-0.12, 0.46) | **0.62 (0.11, 1.1)** | **0.46 (-0.00, 0.93)** | 0.60 (0.31, 0.89) | 0.53 (0.21, 0.85) | **0.75 (0.42, 1.1)** | **1.1 (0.69, 1.6)** | 1.6 (0.77, 2.5) | -0.20 (-0.78, 0.38) | **0.33 (-0.02, 0.67)** |
| Asian/PI | **14.2 (-168.4, 196.9)** | -0.05 (-0.52, 0.43) | **-0.52 (-1.4, 0.31)** | **-0.23 (-0.98, 0.52)** | 0.51 (0.04, 0.98) | 0.24 (-0.28, 0.77) | **0.20 (-0.32, 0.73)** | **0.18 (-0.54,0.89)** | 0.76 (-0.68, 2.2) | -0.07 (-1.0, 0.88) | **-0.08 (-0.64, 0.49)** |
| Hispanic | **54.3 (-63.4, 172.0)** | -0.03 (-0.33, 0.28) | **0.19 (-0.35, 0.74)** | **-0.28 (-0.77, 0.21)** | 0.18 (-0.13, 0.49) | 0.36 (0.02, 0.71) | **-0.04 (-0.39, 0.30)** | **0.45 (-0.02,0.92)** | 0.51 (-0.44, 1.5) | -0.27 (-0.89, 0.35) | **0.15 (-0.22, 0.52)** |
| Black | **-117.4 (-230.5, -4.3)** | -0.08 (-0.38, 0.22) | **-0.41 (-0.94, 0.12)** | **-0.48 (-0.96, -0.00)** | 0.11 (-0.30, 0.52) | -0.05 (-0.50, 0.40) | **0.44 (-0.01, 0.89)** | **-0.00 (-0.61,0.61)** | 0.11 (-1.1, 1.3) | -0.02 (-0.63, 0.58) | **-0.28 (-0.64, 0.07)** |

^a^Results correspond to estimated change in neonatal anthropometric measure per 1-unit increase (95% CI) in log-transformed plasma concentration (continuous measures) or among exposed compared to unexposed (cut-point measures) from unadjusted generalized linear regression models, including time to exam only (except birthweight which was performed at birth).

^b^1 site excluded for incorrect calipers.

^c^Excluded: <37 weeks, <2000g (n= 99; outside range validated per formula to calculate %fat); negative and missing values of % fat mass (n=58).

^d^Passive smoker (≥1 ng/mL) vs non-smoker (reference; <1 ng/mL).

^e^≥LOQ vs < LOQ (reference; LOQ_cotinine_ = 0.05 ng/mL).

^f^≥LOQ vs < LOQ (reference; LOQ_nicotine_ = 0.13 ng/mL).

Abbreviations: LOQ, limit of quantification; CI, confidence interval; PI, Pacific Islander.

**BOLD: Statistically significant differences in the association between biomarker concentration and neonatal anthropometric measure by race/ethnicity (*P_interaction_*<0.1).**
