# Supplementary material for: Association between early gestation passive smoke exposure and neonatal size among self-reported non-smoking women by race/ethnicity: A cohort study
Source: PLoS One. 2021 Nov 18;16(11):e0256676. doi: 10.1371/journal.pone.0256676 (PMC8601432; doi:10.1371/journal.pone.0256676)
Supplement: S4 Table — (DOCX) [file pone.0256676.s007.docx]

**S4 Table. Plasma biomarker concentration-clinical outcomes associations by race/ethnicity among non-smoking pregnant women.^a^**

| **Biomarker** | **Low birthweight (<2500 g)** | | **Macrosomia (>4000 g)** | |
| --- | --- | --- | --- | --- |
|  | **Unadjusted OR (95% CI)** | **Adjusted^b^ OR (95% CI)** | **Unadjusted OR (95% CI)** | **Adjusted^b^ OR (95% CI)** |
| **Cotinine** | | | | |
| White | 0.00 (0.00, 308.8) | 0.00 (0.00, 78.0) | 1.1 (0.87, 1.36) | 1.1 (0.87, 1.3) |
| Asian/PI | 3.1 (1.2, 7.9) | 2.5 (1.0, 6.8) | 2.7 (0.75, 9.5) | 2.0 (0.56, 7.2) |
| Hispanic | 0.93 (0.32, 2.7) | 0.85 (0.27, 2.7) | 1.3 (0.93, 1.7) | 1.2 (0.88, 1.6) |
| Black | 1.15 (1.0, 1.3) | 1.16 (1.0, 1.4) | 0.62 (0.20, 1.6) | 0.74 (0.31, 1.8) |
| **Nicotine** | | | | |
| White | 0.72 (0.31, 1.7) | 0.71 (0.30, 1.7) | 1.1 (0.89, 1.4) | 1.1 (0.86, 1.41) |
| Asian/PI | 0.95 (0.28, 3.3) | 0.90 (0.27, 3.02) | 1.6 (0.73, 3.3) | 1.5 (0.72, 3.05) |
| Hispanic | 1.2 (0.64, 2.2) | 1.14 (0.60, 2.2) | 1.3 (0.98, 1.8) | 1.3 (0.93, 1.7) |
| Black | 1.1 (0.96, 1.3) | 1.1 (0.95, 1.3) | 0.82 (0.47, 1.4) | 0.86 (0.49, 1.5) |

^a^Based on logistic regression models; each model separately in comparison to normal birthweight group (2500-4000 g); results correspond estimated plasma biomarker-clinical outcome association within each racial/ethnic group.

^b^Adjusted for maternal age, infant sex, maternal height, weight, education, and parity.

Abbreviations: OR, odds ratio; CI, confidence interval; PI, Pacific Islander.
