# Supplementary material for: Association between early gestation passive smoke exposure and neonatal size among self-reported non-smoking women by race/ethnicity: A cohort study
Source: PLoS One. 2021 Nov 18;16(11):e0256676. doi: 10.1371/journal.pone.0256676 (PMC8601432; doi:10.1371/journal.pone.0256676)
Supplement: S5 Table — (DOCX) [file pone.0256676.s008.docx]

**S5 Table. Interaction by race/ethnicity in plasma biomarker concentrations-neonatal anthropometrics association among non-smoking pregnant women^a^ (sensitivity analyses in the standard population^b^).**

| **Neonatal anthropometric measure** | **Continuous plasma concentration^c^** | | **Categorical** | | |
| --- | --- | --- | --- | --- | --- |
|  | **Cotinine** | **Nicotine** | **Smoking per cotinine^d^** | **LOQ_cotinine_^e^** | **LOQ_nicotine_^f^** |
| **Unadjusted model^g^** | | | | | |
| *Non-skeletal measures* | | | | | |
| Birthweight | 0.456 | 0.125 | 0.355 | 0.565 | 0.141 |
| Mid-upper arm circumference | 0.709 | **0.094** | 0.210 | 0.280 | 0.671 |
| Abdominal circumference | 0.554 | 0.166 | 0.198 | 0.782 | 0.328 |
| Mid-upper thigh circumference | 0.551 | **0.073** | 0.236 | 0.285 | **0.077** |
| Subscapular skinfold | 0.283 | 0.333 | 0.557 | 0.253 | 0.531 |
| Triceps skinfold | 0.364 | 0.146 | 0.591 | 0.290 | 0.225 |
| Abdominal flank skinfold | 0.862 | 0.564 | 0.801 | 0.173 | **0.008** |
| Anterior thigh skinfold | 0.129 | 0.223 | 0.398 | **0.059** | **0.033** |
| Percent fat mass | 0.403 | 0.383 | 0.297 | 0.143 | 0.109 |
| *Skeletal measures* | | | | | |
| Exam length | 0.918 | 0.325 | 0.573 | 0.117 | 0.620 |
| Head circumference | 0.725 | 0.280 | 0.938 | 0.456 | 0.103 |
| **Adjusted model^h^** | | | | | |
| *Non-skeletal measures* | | | | | |
| Birthweight | 0.6101 | 0.2065 | 0.2887 | 0.6023 | 0.2193 |
| Mid-upper arm circumference | 0.8011 | 0.1745 | 0.1698 | 0.4166 | 0.7982 |
| Abdominal circumference | 0.5386 | 0.2134 | 0.1664 | 0.8399 | 0.4351 |
| Mid-upper thigh circumference | 0.6754 | 0.1490 | 0.2553 | 0.2802 | 0.1090 |
| Subscapular skinfold | 0.3516 | 0.3911 | 0.5927 | 0.2459 | 0.6682 |
| Triceps skinfold | 0.4543 | 0.2284 | 0.5528 | 0.2681 | 0.2830 |
| Abdominal flank skinfold | 0.8666 | 0.6708 | 0.7999 | 0.2369 | **0.0101** |
| Anterior thigh skinfold | 0.1537 | 0.2894 | 0.3868 | **0.0676** | **0.0640** |
| Percent fat mass | 0.5046 | 0.4623 | 0.3205 | 0.2569 | 0.1596 |
| *Skeletal measures* | | | | | |
| Exam length | 0.9054 | 0.2619 | 0.5212 | **0.0556** | 0.5232 |
| Head circumference | 0.8482 | 0.5152 | 0.8167 | 0.2733 | 0.2282 |

^a^Based on generalized linear models, values reported in table are p-values from Type III sums of squares for biomarker x race/ethnicity interaction term (*P_interaction_*; global); *P_interaction_*<0.1 considered statistically significant.

^b^Live-birth, term delivery ≥37 weeks, did not develop pregnancy-related complications, without fetal anomalies.

^c^Plasma concentrations of cotinine and nicotine (ng/mL) were log-transformed (log[1+value]) then rescaled by their standard deviation for analysis.

^d^Passive smoker (≥1 ng/mL) vs non-smoker (reference; <1 ng/mL).

^e^≥LOQ vs < LOQ (reference; LOQ_cotinine_ = 0.05 ng/mL).

^f^≥LOQ vs < LOQ (reference; LOQ_nicotine_ = 0.13 ng/mL).

^g^Adjusted for time to exam only (except birthweight which was performed at birth).

^h^Adjusted for maternal age, infant sex, maternal height, weight, education, parity, and time to exam (except birthweight which was performed at birth).

Abbreviations: LOQ, limit of quantification.

**BOLD: Statistically significant differences in the association between biomarker concentration and neonatal anthropometric measure by race/ethnicity (*P_interaction_*<0.1).**
