# Supplementary material for: Association between early gestation passive smoke exposure and neonatal size among self-reported non-smoking women by race/ethnicity: A cohort study
Source: PLoS One. 2021 Nov 18;16(11):e0256676. doi: 10.1371/journal.pone.0256676 (PMC8601432; doi:10.1371/journal.pone.0256676)
Supplement: S6 Table — (DOCX) [file pone.0256676.s009.docx]

**S6 Table. Continuous plasma biomarker concentration-neonatal anthropometrics associations by race/ethnicity in standard population of non-smoking pregnant women.^a^**

| **Biomarker and smoking status** | **Non-skeletal measures** | | | | | | | | | **Skeletal measures (cm; n=1593)** | |
| --- | --- | --- | --- | --- | --- | --- | --- | --- | --- | --- | --- |
|  | **Birthweight (g; n=1676)** | **Circumferences (cm; n=1593)** | | | **Skinfolds (mm; n=1496)^b^** | | | | **Percent fat mass (n=1445)^c^** | **Exam length** | **Head circumfer-ence** |
|  |  | **Mid-upper arm** | **Abdominal** | **Mid-upper thigh** | **Subscap-ular** | **Triceps** | **Abdominal flank** | **Anterior thigh** |  |  |  |
| **Nicotine_unadj_^d^** | | | | | | | | | | | |
| White | 210.8 (-18.6, 440.3) | **0.47 (-0.20, 1.1)** | 0.94 (-0.18, 2.1) | **0.65 (-0.42, 1.7)** | 1.1 (0.44, 1.8) | 1.2 (0.43, 2.0) | 1.2 (0.43, 2.0) | 2.1 (1.0, 3.1) | 3.1 (0.99, 5.1) | -0.84 (-2.1, 0.47) | 0.39 (-0.38, 1.2) |
| Asian/PI | -111.7 (-596.3, 372.9) | **-1.1 (-2.6, 0.28)** | -0.45 (-2.8, 1.9) | **-0.86 (-3.1, 1.4)** | 1.1 (-0.33, 2.6) | 0.07 (-1.6, 1.7) | 0.84 (-0.82, 2.5) | 0.98 (-1.3, 3.2) | 2.9 (-1.5, 7.3) | -2.0 (-4.8, 0.75) | -0.17 (-1.8, 1.5) |
| Hispanic | -91.5 (-408.9, 225.9) | **-0.56 (-1.5, 0.37)** | -0.07 (-1.6, 1.5) | **-1.3 (-2.8, 0.16)** | 0.58 (-0.38, 1.5) | 0.44 (-0.64, 1.5) | 0.24 (-0.86, 1.3) | 1.3 (-0.20, 2.8) | 0.69 (-2.2, 3.6) | -1.4 (-3.2, 0.41) | 0.36 (-0.71, 1.4) |
| Black | -110.3 (-249.9, 29.4) | **-0.31 (-0.72, 0.09)** | -0.55 (-1.2, 0.13) | **-0.88 (-1.5, -0.23)** | 0.19 (-0.61, 1.00) | -0.14 (-1.0, 0.76) | 1.0 (0.10, 1.9) | 0.35 (-0.88, 1.6) | 0.68 (-1.7, 3.1) | -0.08 (-0.87, 0.72) | -0.40 (-0.86, 0.07) |
| **Nicotine_adj_^e^** | | | | | | | | | | | |
| White | 189.5 (-32.0, 411.0) | 0.37 (-0.30, 1.0) | 0.79 (-0.33, 1.9) | 0.34 (-0.72, 1.4) | 0.97 (0.28, 1.7) | 1.0 (0.28, 1.8) | 1.1 (0.34, 1.9) | 1.8 (0.73, 2.8) | 2.7 (0.63, 4.7) | -0.73 (-2.0, 0.54) | 0.34 (-0.40, 1.1) |
| Asian/PI | -12.5 (-477.9, 452.8) | -1.0 (-2.4, 0.38) | -0.08 (-2.4, 2.3) | -0.74 (-3.0, 1.5) | 1.2 (-0.27, 2.6) | 0.16 (-1.5, 1.8) | 0.95 (-0.69, 2.6) | 1.0 (-1.2, 3.2) | 3.6 (-0.76, 7.9) | -1.6 (-4.2, 1.1) | 0.19 (-1.4, 1.8) |
| Hispanic | -69.8 (-374.7, 235.0) | -0.58 (-1.5, 0.35) | 0.02 (-1.5, 1.6) | -1.3 (-2.8, 0.14) | 0.62 (-0.34, 1.6) | 0.44 (-0.63, 1.5) | 0.30 (-0.79, 1.4) | 1.3 (-0.12, 2.8) | 0.96 (-1.9, 3.8) | -1.5 (-3.2, 0.29) | 0.38 (-0.66, 1.4) |
| Black | -90.0 (-226.1, 46.1) | -0.28 (-0.69, 0.13) | -0.61 (-1.3, 0.07) | -1.0 (-1.7, -0.37) | 0.12 (-0.67, 0.92) | -0.18 (-1.1, 0.72) | 1.0 (0.10, 1.9) | 0.21 (-1.0, 1.4) | 0.67 (-1.7, 3.0) | 0.11 (-0.67, 0.89) | -0.22 (-0.68, 0.23) |
| **Cotinine**_unadj_^d^ | | | | | | | | | | | |
| White | 22.5 (-67.8, 112.9) | 0.14 (-0.12, 0.40) | 0.16 (-0.28, 0.60) | 0.10 (-0.32, 0.52) | 0.24 (-0.03, 0.50) | 0.26 (-0.04,0.56) | 0.07 (-0.24,0.38) | 0.44 (0.03, 0.86) | 0.34 (-0.46, 1.1) | -0.17 (-0.68, 0.34) | 0.02 (-0.28, 0.32) |
| Asian/PI | -97.5 (-521.1, 326.1) | -0.16 (-1.4, 1.1) | -1.3 (-3.4, 0.84) | -0.53 (-2.6, 1.5) | -0.54 (-1.8, 0.76) | -0.35 (-1.8, 1.1) | 0.06 (-1.4, 1.5) | 0.33 (-1.7, 2.3) | -2.1 (-6.0, 1.8) | 0.38 (-2.1, 2.8) | -0.52 (-2.0, 0.93) |
| Hispanic | 16.4 (-148.3, 181.1) | -0.04 (-0.51, 0.44) | 0.12 (-0.68, 0.91) | 0.16 (-0.61, 0.92) | -0.04 (-0.53, 0.45) | 0.11 (-0.44,0.66) | -0.21 (-0.77,0.35) | -0.13 (-0.89, 0.62) | -0.39 (-1.9, 1.1) | 0.11 (-0.82, 1.0) | -0.09 (-0.63, 0.46) |
| Black | -56.4 (-110.3, -2.4) | -0.04 (-0.19, 0.12) | -0.04 (-0.30, 0.22) | -0.20 (-0.45, 0.05) | -0.07 (-0.31, 0.16) | -0.08 (-0.34,0.18) | 0.00 (-0.27,0.27) | -0.20 (-0.56, 0.16) | -0.38 (-1.1, 0.32) | 0.00 (-0.30, 0.31) | -0.16 (-0.34, 0.02) |
|  |  |  |  |  |  |  |  |  |  |  |  |
|  |  |  |  |  |  |  |  |  |  |  |  |
|  |  |  |  |  |  |  |  |  |  |  |  |
|  |  |  |  |  |  |  |  |  |  |  |  |
|  |  |  |  |  |  |  |  |  |  |  |  |
| **Biomarkerand smoking status** | **Non-skeletal measures** | | | | | | | | | **Skeletal measures**  **(cm; n=1593)** | |
|  | **Birthweight (g; n=1676)** | **Circumferences (cm; n=1593)** | | | **Skinfolds (mm; n=1496)^b^** | | | | **Percent fat mass (n=1445)^c^** | **Exam length** | **Head circumfer-ence** |
|  |  | **Mid-upper arm** | **Abdominal** | **Mid-upper thigh** | **Subscap-ular** | **Triceps** | **Abdominal flank** | **Anterior thigh** |  |  |  |
| **Cotinine**_adj_^e^ | | | | | | | | | | | |
| White | 29.0 (-58.0, 115.9) | 0.13 (-0.13, 0.40) | 0.15 (-0.29, 0.58) | 0.04 (-0.37, 0.46) | 0.21 (-0.05, 0.48) | 0.23 (-0.07, 0.53) | 0.07 (-0.24, 0.37) | 0.40 (-0.01, 0.81) | 0.35 (-0.45, 1.1) | -0.12 (-0.62, 0.37) | 0.02 (-0.27, 0.31) |
| Asian/PI | -77.2 (-487.3, 332.8) | -0.20 (-1.5, 1.1) | -1.4 (-3.5, 0.73) | -0.71 (-2.7, 1.3) | -0.48 (-1.8, 0.82) | -0.41 (-1.9, 1.0) | 0.16 (-1.3, 1.6) | 0.48 (-1.5, 2.5) | -1.7 (-5.6, 2.1) | 0.19 (-2.2, 2.6) | -0.54 (-2.0, 0.87) |
| Hispanic | 8.8 (-149.8, 167.3) | -0.06 (-0.53, 0.42) | 0.10 (-0.69, 0.89) | 0.04 (-0.71, 0.80) | -0.07 (-0.56, 0.41) | 0.02 (-0.52, 0.57) | -0.20 (-0.75, 0.36) | -0.20 (-0.95, 0.55) | -0.38 (-1.8, 1.1) | 0.11 (-0.79, 1.0) | -0.09 (-0.62, 0.44) |
| Black | -37.2 (-90.0, 15.6) | 0.00 (-0.16, 0.16) | -0.02 (-0.28, 0.24) | -0.21 (-0.46, 0.05) | -0.07 (-0.30, 0.17) | -0.07 (-0.33, 0.19) | 0.03 (-0.23, 0.30) | -0.20 (-0.55, 0.16) | -0.29 (-0.99, 0.40) | 0.09 (-0.22, 0.39) | -0.08 (-0.25, 0.10) |

^a^Results correspond to estimated change in neonatal anthropometric measure per 1-unit increase (95% CI) in log-transformed plasma concentration in generalized linear models; standard population: live-birth, term delivery ≥37 weeks, did not develop pregnancy-related complications, without fetal anomalies.

^b^1 site excluded for incorrect calipers (n=97).

^c^Excluded:<37weeks, <2000g (n=4; outside range validated per formula to calculate %fat); negative and missing values of % fat mass (n=47).

^d^Adjusted for time to exam only (except birthweight which was performed at birth).

^e^Adjusted for maternal age, infant sex, maternal height, weight, education, parity, and time to exam (except birthweight which was performed at birth).

Abbreviations: CI, confidence interval; PI, Pacific Islander.

**BOLD: Statistically significant differences in the association between biomarker concentration and neonatal anthropometric measure by race/ethnicity (*P_interaction_*<0.1).**
