# Supplementary material for: Association between early gestation passive smoke exposure and neonatal size among self-reported non-smoking women by race/ethnicity: A cohort study
Source: PLoS One. 2021 Nov 18;16(11):e0256676. doi: 10.1371/journal.pone.0256676 (PMC8601432; doi:10.1371/journal.pone.0256676)
Supplement: S7 Table — (DOCX) [file pone.0256676.s010.docx]

**S7 Table. Plasma biomarker concentration cut-points-neonatal anthropometrics associations by race/ethnicity in standard population of non-smoking pregnant women.^a^**

| **Biomarker and smoking status** | **Non-skeletal measures** | | | | | | | | | **Skeletal measures (cm; n=1593)** | |
| --- | --- | --- | --- | --- | --- | --- | --- | --- | --- | --- | --- |
|  | **Birthweight (g; n=1676)** | **Circumferences (cm; n=1593)** | | | **Skinfolds (mm; n=1496)^b^** | | | | **Percent fat mass (n=1445)^c^** | **Exam length** | **Head circumference** |
|  |  | **Mid-upper arm** | **Abdominal** | **Mid-upper thigh** | **Subscapular** | **Triceps** | **Abdominal flank** | **Anterior thigh** |  |  |  |
| **Cotinine** | | | | | | | | | | | |
| *Any smoking_unadj_^d,e^* | | | | | | | | | | | |
| White | -125.4 (-436.5, 185.6) | 0.45 (-0.45, 1.4) | -0.01 (-1.5, 1.5) | 0.22 (-1.2, 1.7) | 0.28 (-0.65, 1.2) | 0.02 (-1.0, 1.1) | -0.20 (-1.3, 0.86) | 0.61 (-0.81, 2.0) | -0.20 (-3.0, 2.6) | -1.0 (-2.8, 0.74) | -0.34 (-1.4, 0.69) |
| Asian/PI | -856.1 ( -1674, -37.9) | -2.3 (-4.6, 0.11) | -4.5 (-8.5, -0.58) | -3.8 (-7.6, -0.01) | -1.2 (-3.7, 1.2) | -1.8 (-4.5, 0.95) | 0.07 (-2.7, 2.8) | -1.9 (-5.6, 1.8) | -6.9 (-14.2, 0.39) | -1.2 (-5.8, 3.4) | -1.1 (-3.8, 1.6) |
| Hispanic | -33.5 (-612.4, 545.4) | -0.12 (-1.8, 1.6) | -0.48 (-3.3, 2.3) | 0.63 (-2.1, 3.3) | -0.42 (-2.1, 1.3) | 0.31 (-1.6, 2.2) | -1.1 (-3.0, 0.91) | -1.5 (-4.1, 1.1) | -3.0 (-8.1, 2.2) | 0.79 (-2.5, 4.0) | -0.11 (-2.0, 1.8) |
| Black | -103.3 (-251.7, 45.2) | 0.20 (-0.24, 0.63) | -0.18 (-0.90, 0.54) | -0.35 (-1.0, 0.34) | -0.38 (-1.0, 0.28) | 0.18 (-0.57, 0.92) | 0.00 (-0.76, 0.76) | -0.20 (-1.2, 0.82) | -0.43 (-2.5, 1.6) | 0.21 (-0.64, 1.1) | -0.23 (-0.72, 0.27) |
| *Any smoking_adj_^e,f^* | | | | | | | | | | | |
| White | -95.4 (-394.3, 203.6) | 0.48 (-0.41, 1.4) | 0.00 (-1.5, 1.5) | 0.14 (-1.3, 1.6) | 0.26 (-0.65, 1.2) | -0.01 (-1.0, 1.0) | -0.17 (-1.2, 0.88) | 0.57 (-0.84, 2.0) | -0.07 (-2.8, 2.7) | -0.86 (-2.6, 0.85) | -0.31 (-1.3, 0.69) |
| Asian/PI | -836.2 ( -1622, -50.1) | -2.3 (-4.6, 0.09) | -4.6 (-8.5, -0.70) | -3.9 (-7.6, -0.12) | -1.2 (-3.6, 1.2) | -1.9 (-4.6, 0.84) | 0.22 (-2.5, 3.0) | -1.8 (-5.5, 1.9) | -6.3 (-13.5, 0.81) | -1.4 (-5.8, 3.1) | -1.2 (-3.8, 1.4) |
| Hispanic | -76.4 (-633.7, 481.0) | -0.21 (-1.9, 1.5) | -0.53 (-3.3, 2.2) | 0.30 (-2.4, 3.0) | -0.49 (-2.2, 1.2) | 0.02 (-1.9, 1.9) | -0.98 (-2.9, 0.96) | -1.7 (-4.3, 0.95) | -2.9 (-7.9, 2.2) | 0.72 (-2.5, 3.9) | -0.24 (-2.1, 1.6) |
| Black | -48.2 (-192.6, 96.3) | 0.31 (-0.12, 0.75) | -0.11 (-0.83, 0.61) | -0.30 (-0.99, 0.39) | -0.34 (-0.99, 0.32) | 0.20 (-0.54, 0.94) | 0.05 (-0.71, 0.80) | -0.12 (-1.1, 0.90) | -0.24 (-2.3, 1.8) | 0.40 (-0.44, 1.2) | -0.03 (-0.52, 0.45) |
| *≥LOQ_unadj_^d,g^* | | | | | | | | | | | |
| White | 9.7 (-113.7, 133.0) | 0.11 (-0.25, 0.48) | -0.13 (-0.73, 0.48) | 0.06 (-0.52, 0.64) | -0.20 (-0.58, 0.18) | 0.11 (-0.31, 0.54) | -0.08 (-0.51, 0.35) | **0.22 (-0.36, 0.80)** | -0.17 (-1.3, 0.97) | -0.45 (-1.2, 0.26) | 0.02 (-0.40, 0.44) |
| Asian/PI | 35.3 (-91.4, 162.1) | 0.25 (-0.14, 0.64) | 0.28 (-0.36, 0.93) | 0.51 (-0.10, 1.1) | -0.31 (-0.71, 0.09) | 0.28 (-0.16, 0.72) | -0.27 (-0.72, 0.19) | **0.29 (-0.31, 0.90)** | -0.20 (-1.4, 0.99) | 0.07 (-0.67, 0.81) | -0.01 (-0.45, 0.43) |
| Hispanic | -26.4 (-136.3, 83.6) | 0.07 (-0.27, 0.40) | 0.19 (-0.38, 0.75) | -0.10 (-0.63, 0.44) | 0.18 (-0.17, 0.52) | 0.22 (-0.17, 0.61) | 0.33 (-0.06, 0.73) | **0.74 (0.20, 1.3)** | 0.83 (-0.21, 1.9) | -0.60 (-1.2, 0.06) | -0.39 (-0.77, -0.00) |
| Black | -63.8 (-145.8, 18.2) | -0.16 (-0.41, 0.08) | 0.01 (-0.40, 0.41) | -0.20 (-0.59, 0.19) | 0.02 (-0.28, 0.32) | -0.19 (-0.52, 0.15) | -0.17 (-0.52, 0.17) | **-0.23 (-0.69, 0.22)** | -0.81 (-1.7, 0.10) | 0.29 (-0.19, 0.77) | -0.21 (-0.48, 0.07) |
| **Biomarker and smoking status** | **Non-skeletal measures** | | | | | | | | | **Skeletal measures (cm; n=1593)** | |
|  | **Birthweight (g; n=1676)** | **Circumferences (cm; n=1593)** | | | **Skinfolds (mm; n=1496)^b^** | | | | **Percent fat mass (n=1445)^c^** | **Exam length** | **Head circumference** |
|  |  | **Mid-upper arm** | **Abdominal** | **Mid-upper thigh** | **Subscapular** | **Triceps** | **Abdominal flank** | **Anterior thigh** |  |  |  |
| *≥LOQ_adj_^f,g^* | | | | | | | | | | | |
| White | 48.6 (-71.5, 168.7) | 0.12 (-0.25, 0.49) | -0.10 (-0.71, 0.51) | -0.04 (-0.62, 0.54) | -0.22 (-0.60, 0.16) | 0.10 (-0.32, 0.53) | -0.05 (-0.48, 0.38) | **0.18 (-0.40, 0.77)** | -0.05 (-1.2, 1.1) | **-0.24 (-0.94, 0.46)** | 0.13 (-0.27, 0.54) |
| Asian/PI | 46.0 (-76.0, 168.1) | 0.27 (-0.11, 0.65) | 0.30 (-0.34, 0.94) | 0.50 (-0.11, 1.1) | -0.32 (-0.71, 0.08) | 0.29 (-0.15, 0.73) | -0.26 (-0.71, 0.19) | **0.30 (-0.30, 0.90)** | -0.15 (-1.3, 1.0) | **0.16 (-0.56, 0.88)** | 0.06 (-0.36, 0.49) |
| Hispanic | -24.0 (-129.7, 81.8) | 0.04 (-0.29, 0.38) | 0.16 (-0.40, 0.71) | -0.17 (-0.70, 0.36) | 0.15 (-0.19, 0.49) | 0.18 (-0.20, 0.57) | 0.33 (-0.06, 0.72) | **0.70 (0.17, 1.2)** | 0.76 (-0.26, 1.8) | **-0.61 (-1.2, 0.03)** | -0.37 (-0.74, -0.00) |
| Black | -27.9 (-109.3, 53.5) | -0.10 (-0.35, 0.15) | 0.08 (-0.34, 0.50) | -0.19 (-0.59, 0.21) | 0.03 (-0.27, 0.33) | -0.21 (-0.55, 0.13) | -0.08 (-0.43, 0.27) | **-0.24 (-0.71, 0.22)** | -0.63 (-1.5, 0.28) | **0.45 (-0.03, 0.93)** | -0.08 (-0.36, 0.20) |
| **Nicotine** | | | | | | | | | | | |
| *≥LOQ_unadj_^d,h^* | | | | | | | | | | | |
| White | 115.7 (14.0, 217.4) | 0.09 (-0.21, 0.39) | 0.53 (0.03, 1.0) | **0.32 (-0.16, 0.80)** | 0.50 (0.19, 0.80) | 0.56 (0.21, 0.90) | **0.83 (0.48, 1.2)** | **1.1 (0.66, 1.6)** | 1.8 (0.83, 2.7) | -0.34 (-0.92, 0.24) | 0.23 (-0.11, 0.57) |
| Asian/PI | -28.9 (-202.2, 144.3) | -0.06 (-0.57, 0.45) | -0.15 (-1.0, 0.70) | **-0.24 (-1.1, 0.57)** | 0.31 (-0.21, 0.83) | 0.10 (-0.49, 0.68) | **0.24 (-0.35, 0.84)** | **0.13 (-0.67, 0.93)** | 0.56 (-1.0, 2.2) | -0.09 (-1.1, 0.90) | -0.21 (-0.80, 0.37) |
| Hispanic | 39.7 (-72.4, 151.8) | -0.06 (-0.40, 0.28) | 0.21 (-0.35, 0.77) | **-0.30 (-0.83, 0.24)** | 0.21 (-0.14, 0.55) | 0.27 (-0.11, 0.66) | **-0.08 (-0.48, 0.31)** | **0.35 (-0.18, 0.88)** | 0.27 (-0.76, 1.3) | -0.43 (-1.1, 0.22) | 0.19 (-0.19, 0.57) |
| Black | -54.9 (-165.6, 55.9) | -0.19 (-0.52, 0.14) | -0.09 (-0.64, 0.45) | **-0.59 (-1.1, -0.07)** | 0.16 (-0.27, 0.59) | -0.02 (-0.49, 0.46) | **0.47 (-0.02, 0.96)** | **0.16 (-0.49, 0.81)** | 0.17 (-1.1, 1.5) | 0.13 (-0.51, 0.76) | -0.33 (-0.70, 0.04) |
| *≥LOQ_adj_^f,h^* | | | | | | | | | | | |
| White | 109.8 (11.8, 207.8) | 0.05 (-0.24, 0.35) | 0.48 (-0.02, 0.97) | 0.21 (-0.26, 0.69) | 0.44 (0.14, 0.75) | 0.51 (0.17, 0.85) | **0.82 (0.47, 1.2)** | **1.0 (0.57, 1.5)** | 1.6 (0.73, 2.6) | -0.26 (-0.83, 0.30) | 0.22 (-0.11, 0.54) |
| Asian/PI | -14.4 (-180.6, 151.7) | -0.05 (-0.55, 0.46) | -0.07 (-0.91, 0.78) | -0.20 (-1.0, 0.61) | 0.33 (-0.19, 0.85) | 0.14 (-0.44, 0.72) | **0.27 (-0.32, 0.85)** | **0.16 (-0.63, 0.96)** | 0.68 (-0.90, 2.2) | -0.00 (-0.97, 0.96) | -0.13 (-0.69, 0.44) |
| Hispanic | 51.9 (-55.8, 159.5) | -0.06 (-0.39, 0.28) | 0.25 (-0.30, 0.81) | -0.28 (-0.81, 0.25) | 0.23 (-0.12, 0.57) | 0.30 (-0.09, 0.68) | **-0.07 (-0.46, 0.32)** | **0.38 (-0.14, 0.91)** | 0.35 (-0.68, 1.4) | -0.38 (-1.0, 0.26) | 0.20 (-0.17, 0.57) |
| Black | -37.2 (-143.8, 69.4) | -0.17 (-0.50, 0.15) | -0.09 (-0.63, 0.45) | -0.66 (-1.2, -0.14) | 0.14 (-0.28, 0.57) | -0.05 (-0.52, 0.43) | **0.45 (-0.03, 0.94)** | **0.11 (-0.54, 0.75)** | 0.12 (-1.2, 1.4) | 0.24 (-0.38, 0.86) | -0.23 (-0.59, 0.13) |

^a^Results correspond estimated change in neonatal anthropometric measure among exposed compared to unexposed (95% CI) from generalized linear regression models; standard population: live-birth, term delivery ≥37 weeks, did not develop pregnancy-related complications, without fetal anomalies.

^b^1 site excluded for incorrect calipers (n=97).

^c^Excluded: <37 weeks, <2000g (n=4; outside range validated per formula to calculate %fat); negative and missing values of % fat mass (n=47).

^d^Adjusted for time to exam only (except birthweight which was performed at birth).

^e^Passive smoker (≥1 ng/mL) vs non-smoker (reference; <1 ng/mL).

^f^Adjusted for maternal age, infant sex, maternal height, weight, education, parity, and time to exam (except birthweight which was performed at birth).

^g^≥LOQ vs < LOQ (reference; LOQ_cotinine_ = 0.05 ng/mL).

^h^≥LOQ vs < LOQ (reference; LOQ_nicotine_ = 0.13 ng/mL).

Abbreviations: LOQ, limit of quantification; CI, confidence interval; PI, Pacific Islander.

**BOLD: Statistically significant differences in the association between biomarker concentration and neonatal anthropometric measure by race/ethnicity (*P_interaction_*<0.1).**
